# Fluocinolone intravitreal implant (Iluvien) for macular edema secondary to immune recovery uveitis in patient with acute myeloid leukemia

**DOI:** 10.1186/s12348-024-00397-y

**Published:** 2024-05-03

**Authors:** JM Cachero Rodríguez, J. Artaraz, Nora Imaz, A. Fonollosa

**Affiliations:** 1 Department of Ophthalmology, Biocruces Bizkaia Health Research Institute, Cruces University Hospital, University of the Basque Country, Barakaldo, Spain; 2 Department of Retina, Instituto Oftalmológico Bilbao, Bilbao, Spain; 3 Instituto Oftalmológico Etxeandia, Galdakao, Spain; 4 Department of Ophthalmology, Donostia University Hospital, Donostia, Spain

**Keywords:** Cytomegalovirus retinitis, Fluocinolone intravitreal implant, Immune recovery uveitis, Uveitis, Cystoid macular edema

## Abstract

**Purpose:**

To report the use of Fluocinolone intravitreal implant (Iluvien) for the treatment of persistent cystoid macular edema (CME) due to immune recovery uveitis syndrome in a patient with previous cytomegalovirus retinitis and acute myeloid leukemia.

**Design:**

Case report.

**Methods:**

The clinical history of a patient who received an Iluvien implant in one eye for the treatment of cystoid macular edema due to immune recovery uveitis syndrome, previously treated with peribulbar Triamcinolone and intravitreal Dexamethasone injections, was reviewed.

**Results:**

A 48-year-old woman presented with cystoid macular edema due to immune recovery uveitis syndrome. The patient had a history of cytomegalovirus retinitis 3.5 years prior, secondary to immunosuppressive treatment for an acute myeloid leukemia. Three periocular triamcinolone injections and two dexamethasone intravitreal implants were performed, but the edema recurred, so fluocinolone intravitreal implant was used, achieving a sustained control of the condition at one year of follow-up.

**Conclusion:**

The Fluocinolone intravitreal implant may be an effective treatment for persistent CME in patients with immune recovery uveitis syndrome.

## Introduction

Cytomegalovirus (CMV) retinitis is one of the most common opportunistic ocular infections in immunocompromised patients. It is characterised by areas of dense whitish retinal infiltration associated with retinal flame hemorrhages, usually starting in the periphery and spreading along the vascular arcades [1]. It is a complication most commonly described in patients with human immunodeficiency virus (HIV), but has also been described in iatrogenically immunosuppressed patients (e.g. recipients of transplants or cancer chemotherapy) [2, 3]. When an immunocompromised patient is effectively treated and the immunity improves, immune recovery syndrome (IRS) may occur, that is, a paradoxical worsening of inflammation in a previously treated opportunistic infection [1–7]. This syndrome has been described both in systemic infectious disease and local ocular disease. Ocular IRS is referred to as immune recovery uveitis (IRU) [1, 8]. In the case of CMV retinitis, speculation on the pathophysiology includes the fact that the intraocular inflammation is a reaction to antigenically altered retinal or glial cells adjacent to the healed CMV lesion or secondary to chronic subclinical viral replication along the border of healed CMV [9]. According to *Nussenblatt et al.*, as immune function improves, a threshold is reached at which the body can mount an intraocular inflammatory response to cytomegalovirus antigens present in the eye. With continued recovery of immune function, a higher threshold is reached at which the immune system inactivates cytomegalovirus, production of antigen stops, and inflammatory reactions subside [10]. Semiology of IRU includes anterior uveitis, vitritis, cystoid macular edema (CME) and epiretinal membranes. Assuming the pathogenic theory of the reaction to subclinical viral replication, the vitritis and anterior uveitis would translate the inflammatory response to the CMV. However, epiretinal membranes and chronic CME may occur as complications of inflammation without active patent vitritis, hence, *sensu stricto*, would be sequelae of IRU.

The Iluvien implant (Fluocinolone Intravitreal Implant, Alimera Alpharetta, Georgia Alimera) provides sustained release of fluocinolone acetonide for up to 36 months. It is implanted by injection through the pars plana into the vitreous. The device has been shown to be effective in controlling intraocular inflammation and reducing the need for systemic and local treatment. Iluvien has been shown to be useful in the treatment of chronic CME in both uveitis and diabetic patients and may help prevent further damage from chronic inflammation.

We describe our experience using Iluvien to treat chronic CME secondary to IRU in a patient with a history of acute myeloid leukemia (AML) and treated CMV retinitis.

### Case

**Fig. 1:**
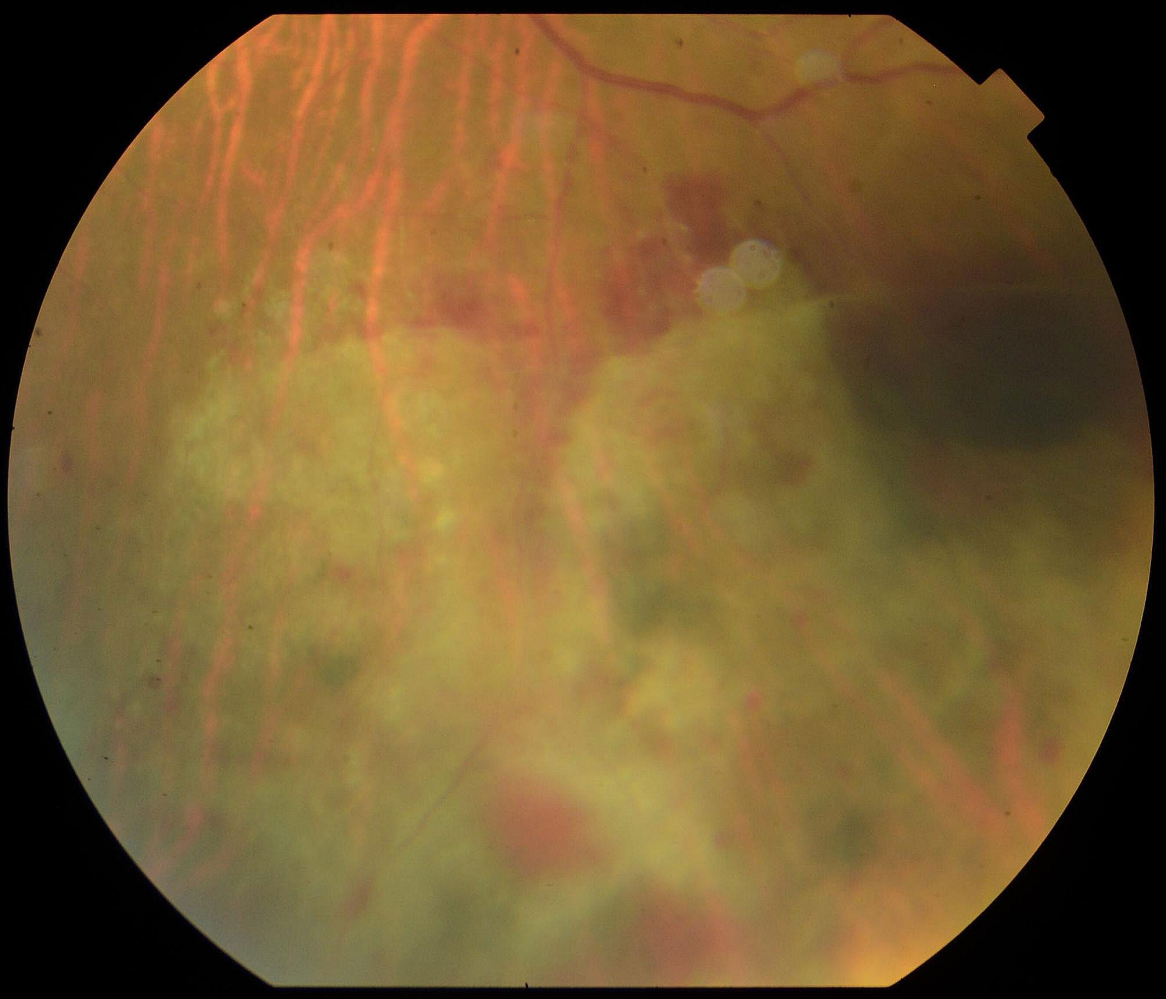
Funduscopy of LE showing inferior hemorrhagic retinitis, suggestive of CMV infection

A 45-year-old woman was diagnosed with AML in 2016. She was treated with allogeneic hematopoietic stem cell transplantation and immunosuppressive drugs. During follow-up, the patient presented with decreased visual acuity (VA) in her left eye. At that time, she was treated with Tacrolimus 2 mg + Mycophenolate Mofetil 2.5 g + Acyclovir 1.6 g + Cotrimoxazole 160/800 mg + Voriconazole 400 mg every 24 h and Prednisone 75 mg every 48 h. On examination, VA (decimal scale) was 1 in the right eye and 0.7 in the left eye (LE); anterior segment biomicroscopy in both eyes showed no inflammation, intraocular pressure (IOP) was 14 mmHg in both eyes. Funduscopy of the LE showed vitritis 1+, inferior hemorrhagic retinitis, consistent with cytomegalovirus (CMV) infection (Fig. 1).

At that time, an aqueous humour sample was obtained which was positive for CMV and treatment with intravitreal injections of Ganciclovir 400 mcg / 0.1 ml + oral Valganciclovir 900 mg every 12 h began. After 5 injections of Ganciclovir and 6 months of follow-up, both intravitreal treatment and Valganciclovir were stopped due to clinical stability. On examination of the LE: VA was 0.8, IOP was 16 mmHg, there was no vitritis and scar foci had developed in the retinitis areas. The systemic CMV viral load was almost undetectable. Two years later, the patient was seen for decreased VA in the LE: VA was 0.5 and a cataract was diagnosed. IOP was 15 mmHg and there was no vitritis. Phacoemulsification and intraocular lens implantation were performed without complications and VA was recovered to 0.8 and IOP was 14 mmHg. At that time, the patient was still on systemic treatment with: Tacrolimus 1.5 mg + Acyclovir 1.6 g + Cotrimoxazole 160/800 mg every 24 h and Prednisone 30 mg every 48 h.

**Fig. 2:**
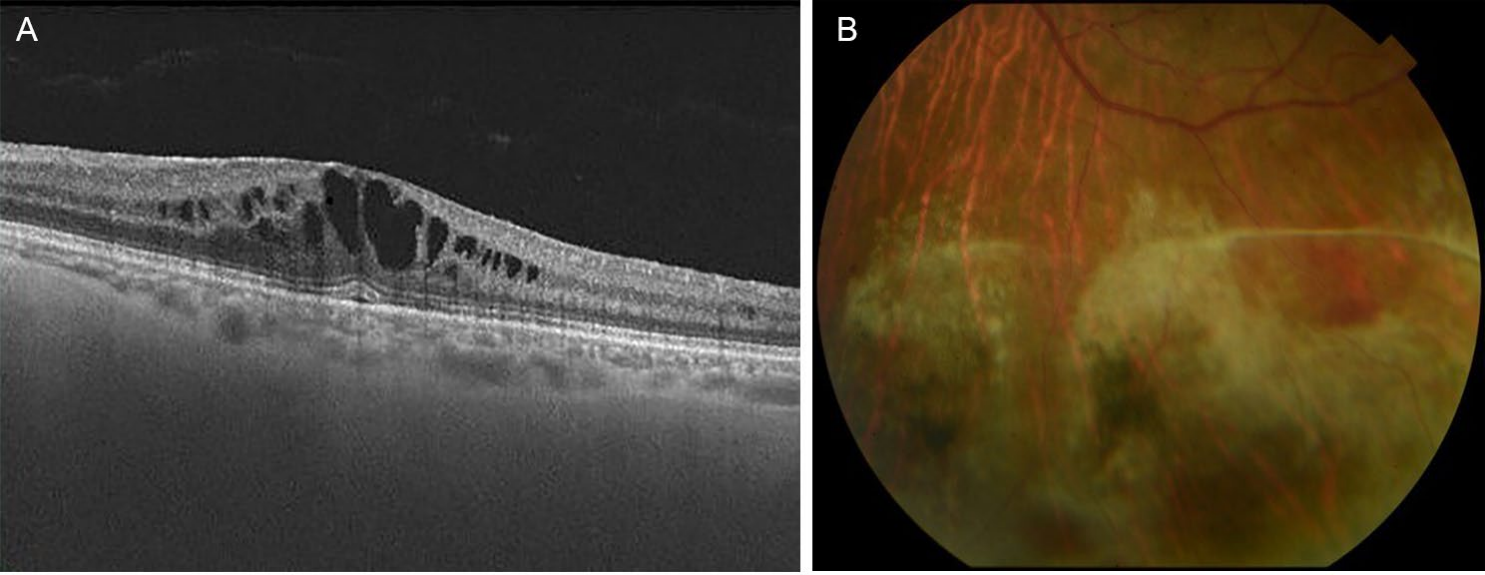
Imaging at IRU syndrome diagnosis. *A*: OCT showing CME. *B*: Retinography showing an area of preretinal fibrosis where retinitis was previously present

One and a half years after surgery, the patient presented with blurred vision in the LE. Systemic treatment at that time was Tacrolimus 1 mg + Acyclovir 1.6 g + Cotrimoxazole 160/800 mg every 24 h. The examination was: VA 0.5, no inflammation in the anterior chamber, IOP 10 mmHg, mild vitritis and CME. In addition, a large area of preretinal fibrosis had developed in the inferior retina *(*Fig. 2*)*.

The diagnosis was IRU in a patient with a history of CMV retinitis. She was treated with 3 injections of peribulbar Triamcinolone (improvement with the first dose, but partial effect with subsequent doses) and oral Acetazolamide, without effect.

**Fig. 3:**
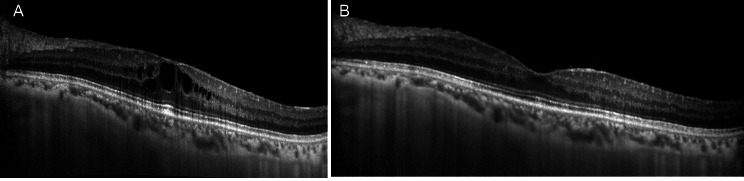
*A*: CME before treatment with Dexamethasone intravitreal implant; *B*: Resolution of the edema after treatment

**Fig. 4:**
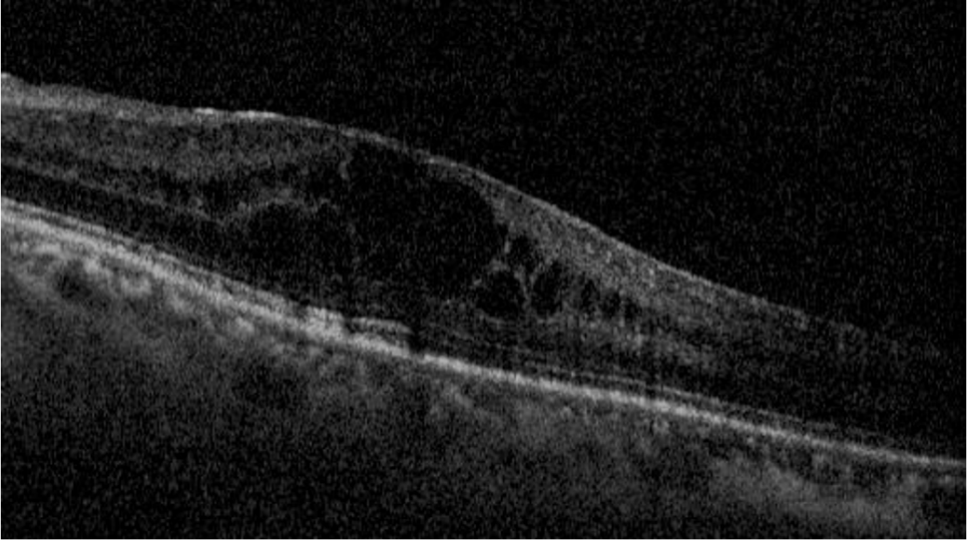
OCT showing recurrence of CME after second injection of Dexamethasone intravitreal implant

Given the lack of improvement, she was treated with intravitreal (ITV) Dexamethasone, which resulted in resolution of the CME *(*Fig. 3*)*, although it recurred after 4 months, so a second ITV Dexamethasone implant was injected. Macular thickness decreased again, but recurred at 4 months (Fig. 4). At this time, the patient was not receiving any immunosuppressive treatment or systemic corticosteroids and was only being treated with oral Acyclovir 1.6 g every 24 h.

**Fig. 5:**
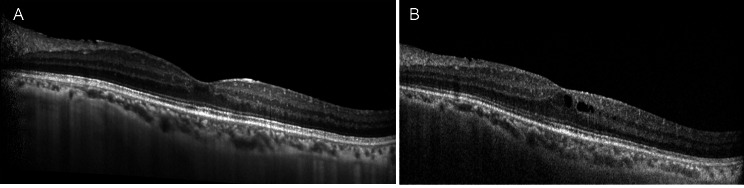
OCT imaging during follow-up after Iluvien implant, *A*: 2 months after treatment, *B*: 1 year after treatment

It was decided to treat the patient with Iluvien (fluocinolone intravitreal implant, Alimera Alpharetta, Georgia), which resulted in resolution of the CME, an effect that has been maintained to date (1 year), though some small microcysts were observed in the OCT. We have not observed signs of reactivation of CMV retinitis during this year (Fig. 5). Oral Acyclovir had been discontinued 2 months prior to implantation.

## Conclusion

CME is an important cause of visual loss in patients with IRU secondary to CMV retinitis [1, 2, 4, 7]. *Kuo et al.* described the characteristics and outcomes of patients with CMV retinitis in the absence of HIV infection. In his series, he compares the risk of developing IRU and the rate of visual loss between immunocompromised patients without HIV infection and those with HIV infection, treated or untreated with highly active antiretroviral therapy (HAART). He concludes that IRU can develop in patients with CMV retinitis but without HIV infection. In their series, IRU occurred after interruption or reduction of immunosuppressive therapy (as in our patient) at a rate similar to that previously reported in patients with HIV infection and CMV retinitis receiving HAART at their institution. The prevalence of visual acuity at or below 20/50 and 20/200 in their series is similar to that in AIDS patients regardless of HAART, demonstrating that CMV retinitis is associated with long term visual morbidity at the time of the diagnosis [2]. 

The management of chronic CME secondary to IRU is challenging. Several reports describe improvement with periocular and intravitreal corticosteroid injections, but the response is transient and often incomplete, without resolution of the CME [2–4]. Many patients require repeated injections for a sustained response, but sometimes a good therapeutic response is not achieved [1]. 

Intravitreal fluocinolone has previously been used to treat CME secondary to IRU, but with Retisert (Bausch and Lomb, Rochester, NY), which is surgically implanted through the pars plana into the vitreous cavity and sutured to the sclera. *Hu et al.* treated CME resulting from IRU in both eyes of two AIDS patients with a history of CMV retinitis with Retisert. In case 1, the patient presented with mild anterior segment inflammation and severe CME, with a VA of 20/200. Maintenance treatment with oral Valganciclovir was also given. Three months after implantation, improvement of inflammation in both eyes and in CME in the left eye was observed, but with no change in VA. In case 2, the patient also presented with bilateral involvement, but more severe in the left eye, where it was decided to perform the implant. The patient did not want to take oral Valganciclovir, so a Ganciclovir implant was placed in the same eye during surgery. Two months later, VA improved from 20/400 to 20/200, and the intraocular inflammation and CME were resolved [4]. 

*Baker et al.* treated an HIV-negative patient with a suspected IRU after allogeneic stem cell transplantation for non-Hodgkin’s lymphoma (NHL). As the immunosuppression waned, he developed severe bilateral ocular involvement: VA counting fingers, severe cataract, vitritis and retinitis with CME. Polymerase chain reaction in the vitreous humour was negative for CMV, and cytology was compatible with a reactive process without evidence of recurrent NHL. She was treated with steroid injection into the floor of the left orbit with little effect. An increase in the dose of Cyclosporine to therapeutic levels resulted in improvement in visual and ophthalmological signs [5]. 

The Iluvien implant has not previously been used to treat CME secondary to IRU. In our case, the treatment was effective. After one year there is no macular edema (though some small microcysts were observed in the last follow-up visit, which may be due to the implant wearing off). Moreover, the treatment was safe: no reactivation of CMV retinitis happened and ocular hypertension, which is one of the main side effects of sustained-release corticosteroid implants, was not observed. We believe it is a good therapeutic option in cases of partial or transient response to periocular or intravitreal corticosteroid injections. We believe it is reasonable, as some authors suggest, that patients receiving local corticosteroids receive prophylactic treatment with antivirals. Moreover, a close follow-up is recommendable due to the well-known risk of infectious uveitis after intravitreal used of steroids [11, 12]. 

## Data Availability

The datasets used and/or analysed during the current study are available from the corresponding author on reasonable request.
